# Serotonin augmentation therapy by escitalopram has minimal effects on amyloid-β levels in early-stage Alzheimer’s-like disease in mice

**DOI:** 10.1186/s13195-017-0298-y

**Published:** 2017-09-12

**Authors:** Christian Ulrich von Linstow, Jonas Waider, Manuela Grebing, Athanasios Metaxas, Klaus Peter Lesch, Bente Finsen

**Affiliations:** 1 Department of Neurobiology, Institute of Molecular Medicine, University of Southern Denmark, J.B. Winsløws Vej 25, 5000 Odense C, Denmark; 2 Molecular Psychiatry, Laboratory of Translational Neuroscience, Department of Psychiatry, Psychosomatics, and Psychotherapy, University of Würzburg, Fuechsleinstrasse 15, 97080 Würzburg, Germany; 3 Laboratory of Psychiatric Neurobiology, Institute of Molecular Medicine, I.M. Sechenov First Moscow State Medical University, Moscow, Russia

**Keywords:** Alzheimer’s disease, APP/PS1, Cerebral amyloidosis, 5-HT, SSRI, 5,7-Dihydroxytryptamine, Cerebrospinal fluid (CSF), Biomarker

## Abstract

**Background:**

Dysfunction of the serotonergic (5-HTergic) system has been implicated in the cognitive and behavioural symptoms of Alzheimer’s disease (AD). Accumulation of toxic amyloid-β (Aβ) species is a hallmark of AD and an instigator of pathology. Serotonin (5-HT) augmentation therapy by treatment with selective serotonin reuptake inhibitors (SSRIs) in patients with AD has had mixed success in improving cognitive function, whereas SSRI administration to mice with AD-like disease has been shown to reduce Aβ pathology. The objective of this study was to investigate whether an increase in extracellular levels of 5-HT induced by chronic SSRI treatment reduces Aβ pathology and whether 5-HTergic deafferentation of the cerebral cortex could worsen Aβ pathology in the APP_swe_/PS1_ΔE9_ (APP/PS1) mouse model of AD.

**Methods:**

We administered a therapeutic dose of the SSRI escitalopram (5 mg/kg/day) in the drinking water of 3-month-old APP/PS1 mice to increase levels of 5-HT, and we performed intracerebroventricular injections of the neurotoxin 5,7-dihydroxytryptamine (DHT) to remove 5-HTergic afferents. We validated the effectiveness of these interventions by serotonin transporter autoradiography (neocortex 79.7 ± 7.6%) and by high-performance liquid chromatography for 5-HT (neocortex 64% reduction). After 6 months of escitalopram treatment or housing after DHT-induced lesion, we evaluated brain tissue by mesoscale multiplex analysis and sections by IHC analysis.

**Results:**

Amyloid-β-containing plaques had formed in the neocortex and hippocampus of 9-month-old APP/PS1 mice after 6 months of escitalopram treatment and 5-HTergic deafferentation. Unexpectedly, levels of insoluble Aβ42 were unaffected in the neocortex and hippocampus after both types of interventions. Levels of insoluble Aβ40 increased in the neocortex of SSRI-treated mice compared with those treated with vehicle control, but they were unaffected in the hippocampus. 5-HTergic deafferentation was without effect on the levels of insoluble/soluble Aβ42 and Aβ40 in both the neocortex and hippocampus. However, levels of soluble amyloid precursor protein α were reduced in the neocortex after 5-HTergic deafferentation.

**Conclusions:**

Because this study shows that modulation of the 5-HTergic system has either no effect or increases levels of insoluble/soluble Aβ42 and Aβ40 in the cerebral cortex of APP/PS1 mice, our observations do not support 5-HT augmentation therapy as a preventive strategy for reducing Aβ pathology.

**Electronic supplementary material:**

The online version of this article (doi:10.1186/s13195-017-0298-y) contains supplementary material, which is available to authorized users.

## Background

Alzheimer’s disease (AD) is a devastating neurodegenerative disorder characterised by amyloid and tau pathology, as well as a progressive decay of brain circuitry, leading to cognitive impairment especially affecting memory [1]. In addition to the cholinergic degenerative damage in AD [2], deterioration of the serotonergic (5-HTergic) system has also attracted attention for its involvement in AD presentation because the 5-HTergic system is involved in the regulation of mood [3] and in memory function [4]. Changes of the 5-HTergic system in AD include loss of raphe neurons [5, 6], reduced activity of tryptophan hydroxylase [7] and a reduction in cortical levels of serotonin (5-HT) [8, 9]. It has been suggested that AD pathology may even originate in the brainstem, which contains the 5-HTergic neurons clustered in the raphe nuclei [10, 11].

The effect of 5-HT augmentation therapy on cognitive function in patients with AD has been examined in placebo-controlled studies with use of selective serotonin reuptake inhibitors (SSRIs) [12–15], but these studies have had limited success [16]. Retrospective cohort studies, however, reported that SSRI treatment reduces cognitive decline and rate of dementia, although not to the level of the general population [17, 18]. Conversely, reducing levels of 5-HT by acute tryptophan depletion worsens cognitive function in patients with AD [19].

As observed in patients with AD [20], ageing APP_SWE_/PS1_ΔE9_ (APP/PS1)-transgenic mice [21] progressively accumulate amyloid-β (Aβ) in their cortex [22] and have reduced density of 5-HTergic fibres and reduced levels of 5-HT [23]. Peroral treatment with 8 mg/kg/day citalopram during a period of 4 months has been reported to reduce Aβ pathology in the neocortex and hippocampus of 7-month-old APP/PS1 mice [24]. Intraperitoneal injection of 5 mg/kg/day paroxetine for 5 months has been shown to retard accumulation of Aβ40 and tau pathology in the hippocampus of 10-month-old triple-transgenic (3 × Tg) mice [25]. A study of amyloid pathology in the hippocampal CA1 region in 3 × Tg mice fed a high-tryptophan diet for 1 month showed reduction of pathology, which was inversely related to the density of 5-HTergic fibres [26]. Conversely, the opposite strategy of inflicting neurotoxin-induced 5-HTergic deafferentation of the forebrain was shown to reduce levels of tau phosphorylation in the neocortex, but not amyloid pathology, in APP/PS1 mice [27].

Given the mixed results of clinical studies and the limited availability of in vivo studies addressing the effect of 5-HT on Aβ pathology in preclinical models of familial AD, we designed this study to investigate the long-term effect of 5-HT augmentation therapy on cerebrospinal fluid (CSF) and brain levels of Aβ in APP/PS1 mice. To this end, one of the most widely used and most serotonin-selective SSRIs, escitalopram, was administered to 3-month-old APP/PS1 mice for a period of 6 months at a therapeutically relevant dosage of 5 mg/kg/day. In addition, neurotoxin-induced degeneration of the 5-HTergic system was used to study the effect of chronic 5-HT reduction on brain levels of Aβ in the APP/PS1 mouse.

After 6 months of treatment with escitalopram, levels of Aβ40 increased in the neocortex of APP/PS1 mice, whereas CSF levels of Aβ were unaffected. 5-HT deafferentation had no effect on any Aβ species, but it did affect levels of soluble amyloid precursor protein α (sAPPα). These results demonstrate that chronic modulation of the 5-HTergic system in prodromal and early AD-like pathology in mice may be inefficient and even contraindicated.

## Methods

### Animals

APP/PS1-Tg mice [21] on a C57BL/6 background were bred and housed in the animal facility at the University of Southern Denmark until they reached 9 months of age. At the age of 3 months, APP/PS1 mice were allocated into groups that were either treated with escitalopram (Cipralex®; Lundbeck A/S, Copenhagen, Denmark) for 6 months or surgically lesioned with 5,7-dihydroxytryptamine (DHT) and housed for 6 months. Additionally, 9-month-old wild-type littermate mice were included along with 6-month-old APP/PS1 mice to evaluate the specificity/sensitivity of the Aβ mesoscale analysis. Mice were kept in a humidity-controlled (45–65%) and temperature-controlled (21 ± 1 °C) environment under a 12:12-h light-dark cycle (lights on at 7 a.m.) with food and water available ad libitum. All experiments were carried out in agreement with the Danish Animal Experiments Inspectorate, Ministry of Environment and Food (2011/562-67 and 2011/561-1950).

### Treatment with escitalopram

Groups of 3-month-old APP/PS1 mice received 5 mg/kg/day escitalopram (Cipralex® 20 mg/ml oral drops; Lundbeck A/S) diluted in normal drinking water to a final concentration of 0.025 mg/ml in their drinking bottles (*n* = 10). To avoid potential modifying effects of light on drug composition, drinking bottles were composed of a black plastic polymer. Vehicle-treated mice received normal drinking water (*n* = 10). The dosage of escitalopram was calculated to result in around 80% occupancy of the serotonin transporter (SERT), which is considered therapeutic in humans [28]. Mice were treated for a period of 6 months, during which their daily water intake and intake of escitalopram and body weight were calculated on the basis of weekly surveillance. The experiment was terminated when mice reached 9 months of age.

### SERT occupancy

Groups of vehicle (*n* = 3) and 1-month-old escitalopram-treated mice (*n* = 3) were killed by cervical dislocation and investigated by autoradiography. The brains were immediately removed and frozen in isopentane on dry ice (−20 °C). Cryostat sections were cut 20 μm thick in the coronal plane using a cryostat (Leica Biosystems, Buffalo Grove, IL, USA) and stored at −80 °C for a period of 1 week. Sections were thawed to room temperature (RT), directly incubated for 5 minutes with 1 nM 3-amino-4-(2-dimethylaminomethylphenylsulfanyl)-benzonitrile ([^3^H]DASB; specific activity 80 Ci/mmol), and then dissolved in 50 mM Tris-HCl buffer containing 150 mM NaCl and 5 mM KCl (pH 7.4). Adjacent sections were incubated under identical conditions in the presence of 10 μM paroxetine to calculate non-specific binding. Sections were next washed in ice-cold 50 mM Tris buffer (twice for 30 seconds each time), dipped in distilled water, dried under a cold stream of air for 2 h and desiccated overnight (O/N) in a box containing silica gel. The sections were placed on Kodak BioMax MR autoradiography film (Carestream Health, Skovlunde, Denmark), which was developed and analysed after 25 days.

### Stereotactic 5-HTergic lesion induced by DHT

The 5-HTergic lesion was induced by intracerebroventricular injection of DHT (5,7-dihydroxytryptamine creatinine sulphate salt, catalogue number 37970; Sigma-Aldrich, Brøndby, Denmark). Three-month-old APP/PS1 mice were randomly distributed into groups of sham- and DHT-lesioned mice (*n* = 10–13/group). Before surgery, Tg mice were administered 25 mg/kg desipramine hydrochloride (catalogue number D3900; Sigma-Aldrich) diluted in sterile PBS i.p. to prevent DHT-induced loss of noradrenergic neurons, then they were anaesthetised by an i.p. injection of a mixture of fentanyl citrate 0.315 mg/ml and fluanisone 10 mg/ml (Hypnorm®; VetaPharma, Leeds, UK) and diazepam 5 mg/ml (Stesolid®; Actavis/Accord Healthcare, Little Island, Ireland) diluted in sterile water. When deeply anaesthetised, mice were placed in a stereotactic frame with an attached microinjection unit (David Kopf Instruments, Tujunga, CA, USA) and injected with 4 μl of a DHT solution (500 mg DHT/ml physiological saline with 0.1% ascorbic acid, catalogue number A4544; Sigma-Aldrich,). Sham-operated mice exclusively received the saline composition. DHT and saline were administered at rates of 1 μl/minute into the left lateral ventricle using a 10-μl syringe (model 801 RN; Hamilton, Reno, NV, USA) with the following coordinates: 1 mm lateral to sagittal suture, 0.5 mm posterior to bregma and 3.0 mm below the dura mater. To avoid spillover, syringes were kept in position for 2 minutes after each injection. After surgery, mice received saline and buprenorphine hydrochloride (Temgesic® 1 μg/20 g diluted in saline; Reckitt Benckiser Healthcare, Hull, UK). Mice were placed in cages located in heated cabinets (28 °C) for 24 h, after which they were transferred to the animal room and kept until the age of 9 months.

### Harvesting of tissue and cerebrospinal fluid

Mice were anaesthetised with a sublethal dose of pentobarbital (Nembutal 0.15 ml/30 g body weight i.p.; Lundbeck) and positioned for CSF removal under a dissection microscope. The skin of the neck was surgically removed, and the dura covering the cisterna magna was exposed. With a thin, pointy glass capillary tube, the dura was punctured, and 1–5 μl of CSF was extracted from the cisterna magna and placed on dry ice before being stored at −80 °C [29]. Next, the brain was removed, and the right hemisphere was isolated for IHC. The neocortex and hippocampus were isolated from the left hemisphere and kept at −80 °C.

### Determination of 5-HT and 5-hydroxyindoleacetic acid

Levels of 5-HT were determined using high-performance liquid chromatography (HPLC) with electrochemical detection essentially as previously described [30]. Briefly, the neocortex and hippocampus were transferred to ice-cold transmitter buffer (150 mM H_3_PO_4_ and 150 μM pentetic acid) and sonicated in three intervals of 10 seconds each with an amplitude set to 15% (tissue dilution 1:20 wt/vol). The homogenate was centrifuged at 36,000 × *g* for 20 minutes at 4 °C, then 40 μl of supernatant was injected into an Agilent 1100 HPLC system (Agilent Technologies, Santa Clara, CA, USA) consisting of an EC 150/4.6 NUCLEODUR 100 3-μm C18 gravity reversed-phase column (Machery-Nagel, Düren, Germany). The electrochemical detector (Machery-Nagel) was adjusted to +0.75 V against an Ag/AgCl reference electrode, and the mobile phase consisted of 16% methanol and 84% phosphate buffer (0.1 M NaH_2_PO_4_, 0.65 mM octanesulfonic acid, 0.5 mM triethylamine and 0.1 mM ethylenediaminetetraacetic acid) adjusted to pH 3.35 with H_3_PO_4_. Detection limits for 5-HT and 5-hydroxyindoleacetic acid (5-HIAA) were 20 pg/mg tissue wet weight.

### Tissue processing and IHC

Isolated hemispheres were fixed in 4% paraformaldehyde (PFA) in Sørensen’s Buffer (SB) for 24 h followed by 1% PFA in SB for an additional 24 h, after which they were dehydrated in graded ethanol and xylene and embedded in paraffin using an HMP 110 tissue processer (MICROM International, Dreieich, Germany). Paraffin-embedded hemispheres were casted into multiblocks and cut into 20-μm-thick sections using a Shandon Finesse ME microtome (Thermo Fisher Scientific, Runcorn, UK). Sections were placed on a water-filled paraffin stretch bath (TFB 35; Medite, Burgdorf, Germany) at a temperature of 45 °C, mounted on microscope slides and dried O/N. Next, sections were incubated for 2 h at 60 °C and stored at 4 °C until use.

Tissue sections were deparaffinised in xylene and rehydrated in graded ethanol before being rinsed in deionised H_2_O. Prior to immunostaining for Aβ, sections were de-masked in 70% formic acid for 30 minutes, followed by rinsing in Tris-buffered saline (TBS) with 1% Triton X-100 (TBS-T) and incubation in TBS with 10% FBS for 30 minutes to block unspecific binding. Biotinylated monoclonal mouse anti-human Aβ antibody (catalogue number BioLegend, San Diego, CA, USA) diluted 1:500 in TBS with 10% FBS was then added O/N at 4 °C. Sections were washed in TBS-T and immersed in TBS/MeOH/H_2_O_2_ (8:1:1) for 10 minutes, and after an additional rinse in TBS-T, they were incubated with horseradish peroxidase-streptavidin (catalogue number RPN1231; GE Healthcare Life Sciences, Brondby, Denmark) diluted 1:200 in TBS with 10% FBS at RT for 3 h. Sections were developed by immersion for 5 minutes in TBS with 3,3′-diaminobenzidine (0.5 mg/ml) and H_2_O_2_ (0.033%) added. After a final TBS rinse, sections were dehydrated in a series of ethanol followed by xylene and then coverslipped with PERTEX (HistoLab Products, Askim, Sweden).

### Meso Scale Discovery multiplex analysis

To determine the content of Aβ40 and Aβ42 in the neocortex and hippocampus of escitalopram-treated and DHT-lesioned APP/PS1 mice, samples were sonicated in ice-cold PBS containing protease and PhosSTOP phosphatase inhibitor cocktail (Roche Diagnostics, Mannheim, Germany). The homogenates were spun at 9000 × *g* for 20 minutes at 4 °C. Supernatants (PBS fraction) were stored at −80 °C, and pellets were resuspended in an 8× volume of 5 M guanidine and 50 mM Tris-HCl buffer. Guanidine and PBS fractions were diluted 2× for analysis employing the V-PLEX panels for Aβ40 and Aβ42 (Aβ peptide panel 1; Meso Scale Discovery, Rockville, MD, USA) and the sAPPα and sw sAPPβ kits (Meso Scale Discovery) in accordance with the manufacturer’s instructions. Plates were processed in a SECTOR Imager 6000 instrument (Meso Scale Discovery), and data were analysed using Discovery Workbench software (Meso Scale Discovery). Values are presented as picograms of Aβ per milligram of total protein.

### Statistics

Data were analysed with Prism version 6 software (GraphPad Software, La Jolla, CA, USA) and are presented as mean ± SEM of 10 animals/group for the SSRI study and 10–13 animals/group for the DHT study. Data from the escitalopram-treated and DHT-lesioned groups were analysed by unpaired, two-tailed Mann-Whitney *U* test. Statistically significant differences are indicated as *p < 0.05, **p < 0.01, ***p < 0.001 and ****p < 0.0001.

## Results

### Dosing confirmation studies

**Fig. 1:**
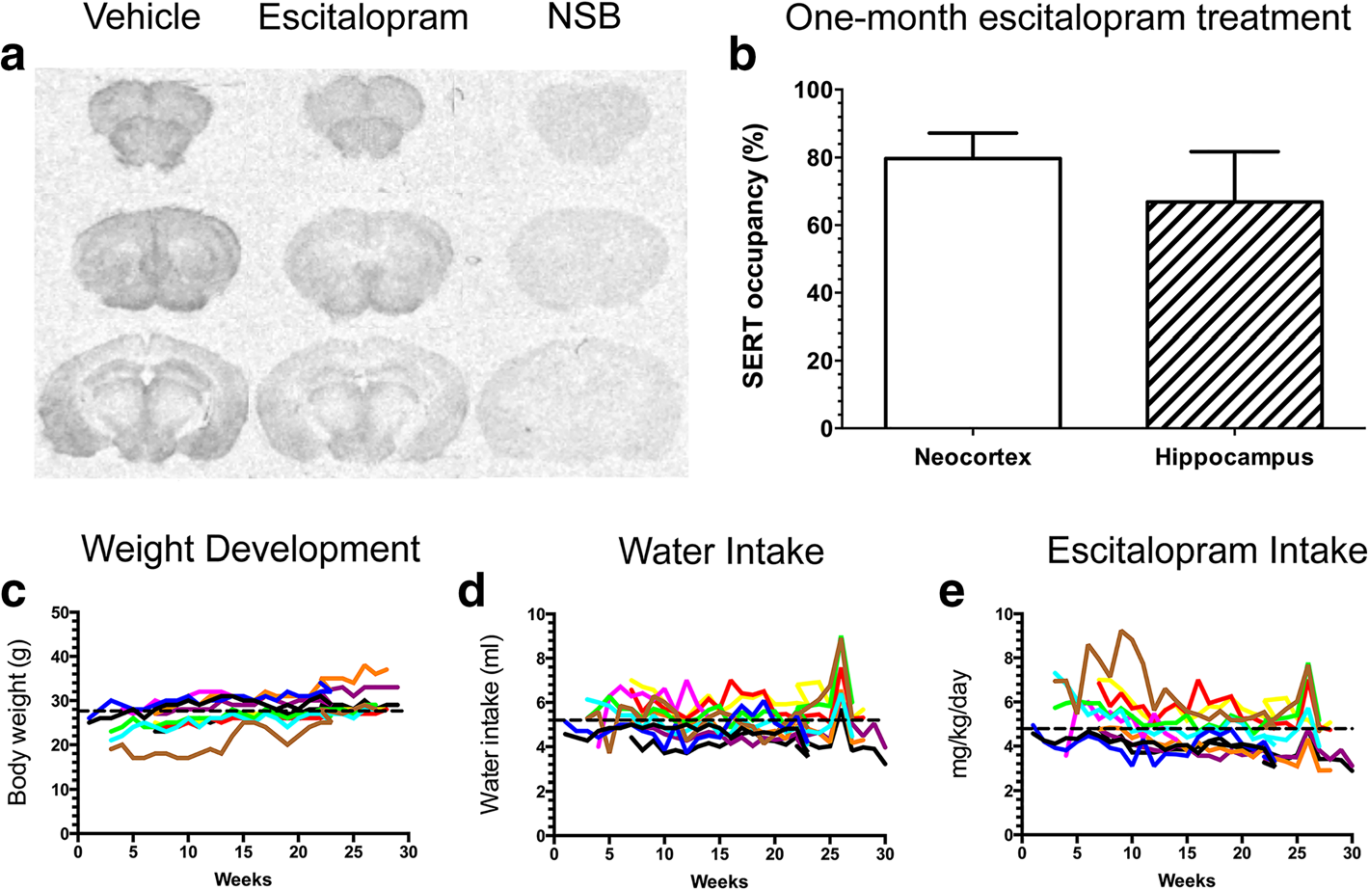
Escitalopram treatment of APP_SWE_/PS1_ΔE9_ (APP/PS1) mice. **a**, **b** Autoradiograms showing the binding of the 3-amino-4-(2-dimethylaminomethylphenylsulfanyl)-benzonitrile radioligand to brain sections from APP/PS1 mice treated with escitalopram (3.83 ± 0.2 mg/kg/day) or vehicle for 1 month, with non-specific binding (NSB) shown to the right (**a**). Graphic presentation showing the serotonin transporter occupancy (as a percentage) in neocortex and hippocampus (**b**). **c–e** Escitalopram intake (**c**), body weight (**d**) and water intake (**e**) in the experimental mice treated with escitalopram from 3 to 9 months of age. The concentration of escitalopram in the drinking water was adjusted to reach 5 mg/kg/day (4.79 ± 0.27 mg/kg/day). Each *coloured line* represents one mouse, and the *dashed line* is the average for all the mice

Given that escitalopram is approximately twice as potent as citalopram [31] and that treatment of APP/PS1 mice with 8 mg/kg/day citalopram per os was previously reported to reduce Aβ load in APP/PS1 mice [24], we aimed to treat animals with escitalopram at a dose of 5 mg/kg/day in the drinking water. To achieve this, the daily water intake of adult APP/PS1 mice was monitored over a period of 4 weeks (*n* = 5; data not shown). Thereafter, the occupancy of SERT following 4 weeks of escitalopram treatment at a nominal dosage of 5 mg/kg/day was determined to confirm dosing effectiveness (*n* = 3) (Fig. 1a, b). The dosage of escitalopram achieved in this study was 3.8 ± 0.2 mg/kg/day (Additional file 1: Figure S1a–c). Autoradiography using the SERT-selective radioligand [^3^H]DASB showed occupancy levels of 79.7 ± 7.6% in the neocortex and 66.9 ± 2.8% in the hippocampus at this dose of escitalopram (Fig. 1a, b). On the basis of these data, we increased the SSRI concentration in the drinking water to achieve a therapeutically relevant intake of 5 mg/kg/day.

### Daily SSRI intake and effects on body weight

Three-month-old APP/PS1 mice were treated with 5 mg/kg/day escitalopram per os for a period of 6 months, whereas vehicle control-treated mice received normal drinking water (*n* = 10/group). Weight development (Fig. 1c), daily water intake (Fig. 1d) and the dosage of escitalopram were monitored throughout the entire experimental period (Fig. 1e). During the experiment, mice weighing 27.7 ± 1.6 g (Fig. 1c) drank, on average, 5.20 ml/day/mouse (Fig. 1d), which corresponded to an intake of escitalopram of 4.79 ± 0.27 mg/kg/day (Fig. 1e). At the end of the experiment, the body weight of 9-month-old APP/PS1 mice receiving escitalopram was comparable to that of vehicle-treated mice (escitalopram 30.5 ± 1.3 g, vehicle 31.4 ± 1.0 g) (Fig. 1c).

### Escitalopram treatment invokes subtle changes of insoluble amyloid-β levels

**Fig. 2:**
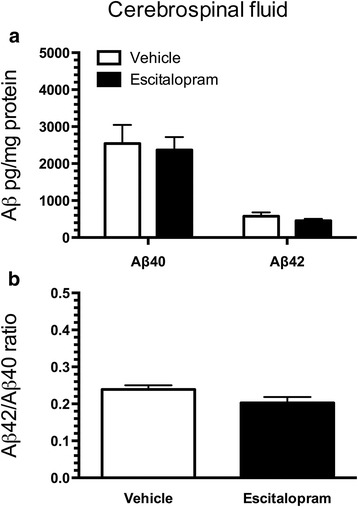
Amyloid-β (Aβ) content in the cerebrospinal fluid (CSF) of APP_SWE_/PS1_ΔE9_ (APP/PS1) mice chronically treated with escitalopram. **a** Content of Aβ40 and Aβ42 and (**b**) the Aβ42/Aβ42 ratio in CSF of APP/PS1 mice treated with 5 mg/kg/day escitalopram from 3 to 9 months of age. Data are presented as mean ± SEM

CSF was drawn immediately prior to the procurement of the neocortex and hippocampus, and the levels of Aβ40 and Aβ42 in the CSF were quantified by multiplex analysis. The levels of Aβ40 and Aβ42 were not different between escitalopram-treated and vehicle-treated APP/PS1 mice (Fig. 2a). There was an almost significant reduction in the CSF ratio of Aβ42/Aβ40 in escitalopram-treated mice compared with vehicle-treated mice (escitalopram 0.20 ± 0.02, vehicle 0.23 ± 0.01) (Fig. 2b). Overall, we detected subtle changes only in levels of Aβ species in the CSF of escitalopram-treated mice compared with vehicle-treated mice.

**Fig. 3:**
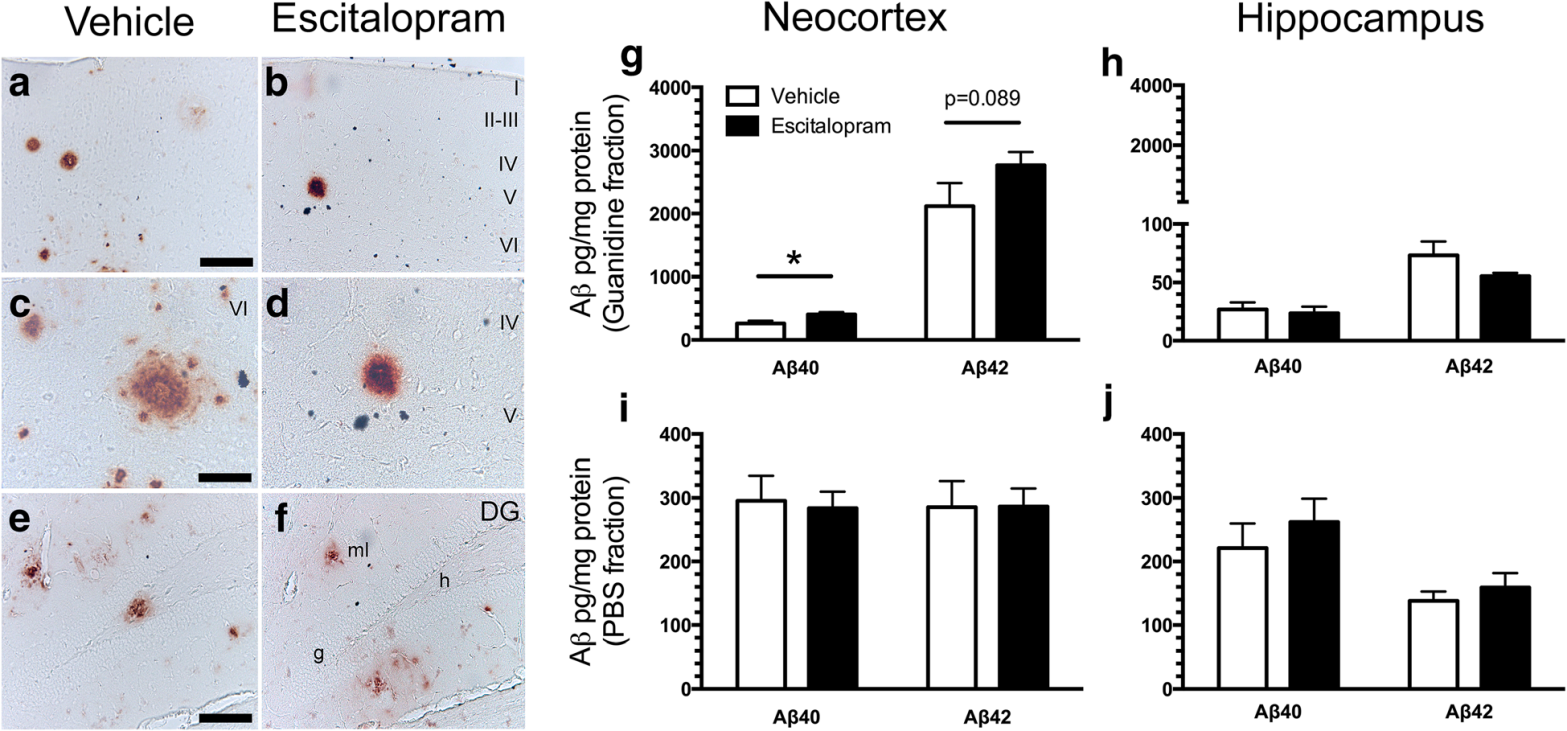
Amyloid-β (Aβ) content in the brain of APP_SWE_/PS1_ΔE9_ (APP/PS1) mice chronically treated with escitalopram. **a–f** Aβ plaques formed in the neocortex (**a**–**d**) and hippocampus (**e** and **f**) of both vehicle-treated (**a**, **c**, **e**) and escitalopram-treated (**b**, **d**, **f**) APP/PS1 mice, shown by 6E10 IHC. Cortical layers are indicated by roman numerals. *DG* Dentate gyrus, *g* Granule cell layer, *h* Hilus, molecular layer. Scale bars = 50 μm (**a**, **b**, **e**, **f**), 100 μm (**c**, **d**). **g** and **h** Levels of insoluble (guanidine fraction) and soluble (PBS fraction) Aβ40 and Aβ42 in the neocortex (**g**, **i**) and hippocampus (**h**, **j**) of APP/PS1 after escitalopram treatment were measured by Meso Scale Discovery multiplex analysis. Data are presented as mean ± SEM and were analysed by unpaired, two-tailed Mann-Whitney *U* test. *p < 0.05

IHC using the 6E10 antibody showed that plaques of human Aβ were abundant in the neocortex and the hippocampus of both escitalopram-treated and vehicle-treated APP/PS1 mice (Fig. 3a–f). In the neocortex, we observed that levels of Aβ40 in the guanidine fraction increased in escitalopram-treated mice compared with vehicle-treated mice (escitalopram 404 ± 38 pg/mg, vehicle 262 ± 43 pg/mg, *p* < 0.05) (Fig. 3g). In the PBS fraction, no differences between escitalopram-treated and vehicle-treated mice were observed (Fig. 3i). Levels of Aβ42 in the cortical guanidine fraction showed a tendency toward an increase in APP/PS1 mice treated with escitalopram compared with vehicle-treated mice (escitalopram 2768 ± 212 pg/mg, vehicle 2119 ± 366 pg/mg, *p* = 0.089) (Fig. 3g), whereas in the PBS fraction, no differences were observed (Fig. 3i). We did not detect any differences in the ratios of Aβ42/Aβ40 in the neocortex (data not shown). None of the Aβ species were detected in the neocortex of wild-type mice (Additional file 2: Figure S2). To support the capacity of the Meso Scale Discovery analysis to detect differences in Aβ levels, we also quantified total Aβ (Aβ38, Aβ40 and Aβ42) levels in the neocortex of 6-month-old APP/PS1 mice and compared the level with that of 9-month-old APP/PS1 mice (Additional file 2: Figure S2). There was a significantly higher level of total Aβ in both the guanidine (*p* < 0.01) and PBS (*p* < 0.001) fractions in 9-month-old compared with 6-month-old APP/PS1 mice (Additional file 2: Figure S2). This is in line with results of a former study done at our laboratory showing a progressive Aβ accumulation with age in the neocortex of APP/PS1 mice [22].

Levels of Aβ40 in the guanidine (Fig. 3h) and PBS (Fig. 3j) fractions of the hippocampus were comparable in escitalopram-treated and vehicle-treated mice. The levels of Aβ42 in the guanidine fraction showed a tendency towards a reduction in mice treated with escitalopram compared with vehicle (escitalopram 55.2 ± 2.7 pg/mg, vehicle 72.8 ± 12 pg/mg, *p* = 0.11) (Fig. 3h).

**Fig. 4:**
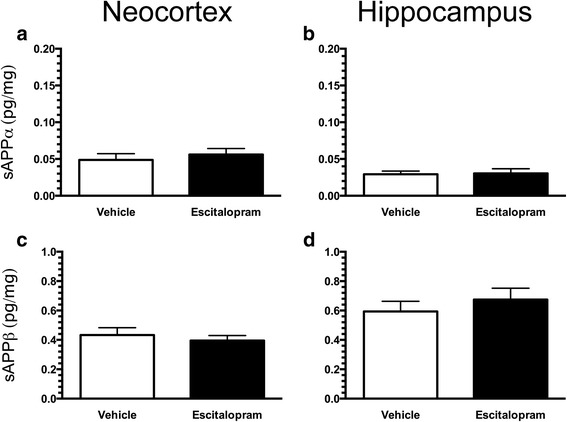
Unchanged levels of soluble amyloid precursor protein α (sAPPα) and sAPPβ in the brain of APP_SWE_/PS1_ΔE9_ (APP/PS1) mice chronically treated with escitalopram. Levels of sAPPα and sAPPβ in the neocortex (**a**, **c**) and hippocampus (**b**, **d**) of APP/PS1 mice after escitalopram treatment were measured by Meso Scale Discovery multiplex analysis. Data are presented as mean ± SEM

To further investigate effects of escitalopram treatment on the processing of APP, we quantified levels of sAPPα and sAPPβ, which are the first cleavage products of APP during amyloidogenesis. We observed no differences between vehicle- and escitalopram-treated APP/PS1 mice in levels of sAPPα in the neocortex (vehicle 0.049 ± 0.009 pg/mg, SSRI 0.056 ± 0.008 pg/mg) (Fig. 4a) or hippocampus (vehicle 0.029 ± 0.004 pg/mg, SSRI 0.030 ± 0.006 pg/mg) (Fig. 4b). In addition, levels of sAPPβ in the neocortex (Fig. 4c) and hippocampus (Fig. 4d) were roughly eight-fold higher than levels of sAPPα and did not differ between groups.

### DHT-induced 5-HTergic deafferentation does not influence amyloid-β levels

Next, we examined if 5-HTergic deafferentation might affect Aβ levels in the brain. Three-month-old APP/PS1 mice were given intracerebroventricular injections of DHT, which induces degeneration of 5-HTergic neurons in the raphe nuclei [27]. DHT-lesioned and sham-operated APP/PS1 mice were allowed to survive until 9 months of age. DHT-lesioned mice appeared unaffected and showed the same body weight as sham-operated mice.

**Fig. 5:**
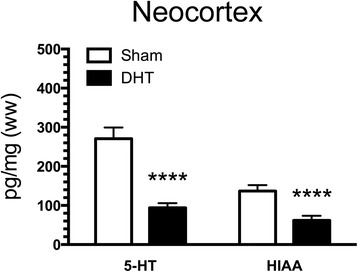
Serotonin (5-HT) deafferentation in APP_SWE_/PS1_ΔE9_ (APP/PS1) mice with 5,7-dihydroxytryptamine (DHT)-induced lesions. Three-month-old APP/PS1 mice were lesioned with DHT and, together with sham-operated mice, allowed to survive until the age of 9 months. Neocortex samples were analysed with high-performance liquid chromatography, and data showed a 60–65% reduction of 5-HT and its metabolite 5-hydroxyindoleacetic acid (5-HIAA). Data are presented as mean ± SEM and were analysed by unpaired, two-tailed Mann-Whitney *U* test. ****p < 0.0001

To evaluate the efficiency of the 5-HTergic deafferentation, tissue samples of neocortex were analysed for the content of 5-HT and its metabolite 5-HIAA (Fig. 5). Levels of 5-HT were reduced by 64% in DHT-lesioned mice (96.73 ± 10.30 pg/ml) compared with sham-operated mice (271.00 ± 28.30 pg/ml) (*p* < 0.0001) (Fig. 5), whereas 5-HIAA decreased by 62% in DHT-lesioned mice (52.40 ± 4.50 pg/ml) compared with sham-operated animals (137.00 ± 15.00 pg/ml) (*p* < 0.0001) (Fig. 5).

**Fig. 6:**
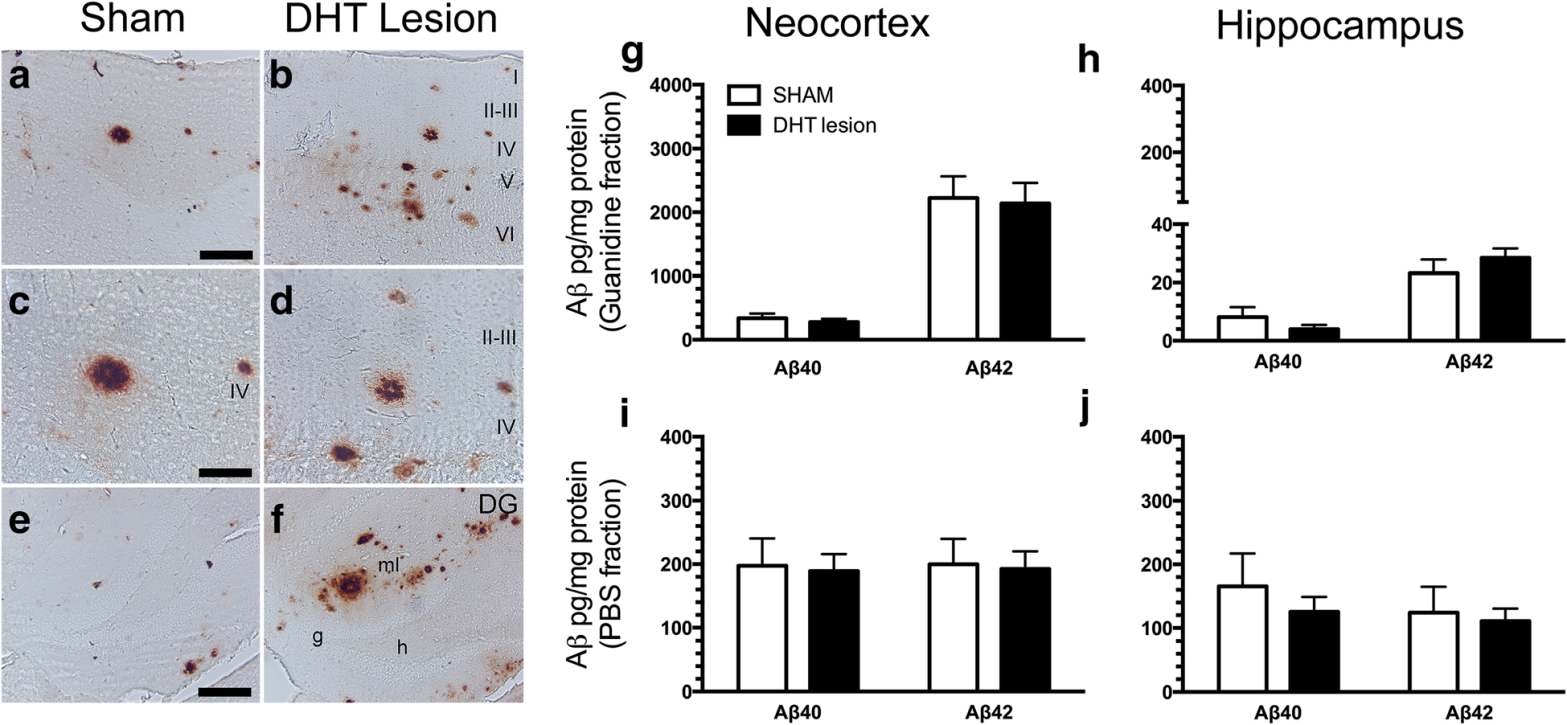
Amyloid-β (Aβ) content in the brain of 5,7-dihydroxytryptamine (DHT)-lesioned APP_SWE_/PS1_ΔE9_ (APP/PS1) mice. **a–f** Aβ plaques formed in the neocortex (**a**–**d**) and hippocampus (**e**, **f**) of sham-operated (**a**, **c**, **e**) and DHT-lesioned (**b**, **d**, **f**) APP/PS1 mice, as shown by shown by 6E10 IHC. Cortical layers are indicated by roman numerals. *DG* Dentate gyrus, *g* Granule cell layer, *h* Hilus, molecular layer. Scale bars = 50 μm (**a**, **b**, **e**, **f**), 100 μm (**c**, **d**). **g–j** Levels of insoluble (guanidine fraction) and soluble (PBS fraction) Aβ40 and Aβ42 in the neocortex (**g**, **i**) and hippocampus (**h**, **j**) of APP/PS1 after sham operation and DHT-induced lesioning were estimated by Meso Scale Discovery multiplex analysis. Data are presented as mean ± SEM

Aβ-containing plaques manifested in the neocortex and hippocampus of both sham-operated and DHT-lesioned mice (Fig. 6a–f). Levels of Aβ40 and Aβ42 in the guanidine and PBS fractions of the neocortex and hippocampus were quantified by multiplex analysis (Fig. 6g–j). We detected no differences in the levels of Aβ40 and Aβ42 in the guanidine and PBS fractions of the neocortex in DHT-lesioned mice compared with sham-operated mice (Fig. 6g, i). Similarly, no differences in the levels of Aβ40 and Aβ42 in the guanidine and PBS fractions of the hippocampus were observed in DHT-lesioned mice compared with sham-operated mice (Fig. 6h, j). No difference in the Aβ42/Aβ40 ratio was detected between DHT-lesioned and sham-operated mice (data not shown).

**Fig. 7:**
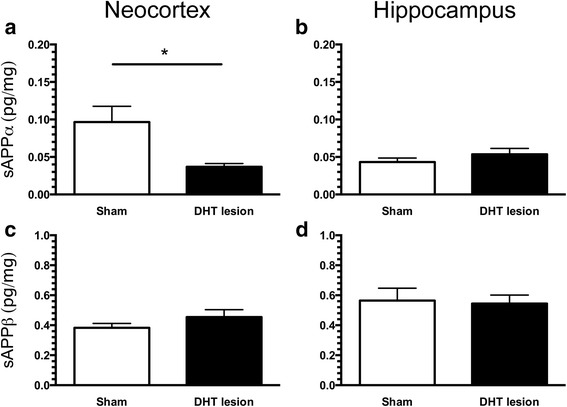
Levels of soluble amyloid precursor protein α (sAPPα) and sAPPβ in the brain of 5,7-dihydroxytryptamine (DHT)-lesioned APP_SWE_/PS1_ΔE9_ (APP/PS1) mice. Levels of sAPPα and sAPPβ in the neocortex (**a**, **c**) and hippocampus (**b**, **d**) of APP/PS1 mice after sham treatment and DHT-induced lesioning were measured by Meso Scale Discovery multiplex analysis. Data are presented as mean ± SEM and were analysed by unpaired, two-tailed Mann-Whitney *U* test. *p < 0.05

Similarly to the previous study employing escitalopram, we also asked whether APP processing would be affected by 5-HTergic deafferentation of the cortex, so we measured levels sAPPα and sAPPβ in the neocortex and hippocampus of sham-operated and DHT-lesioned APP/PS1 mice (Fig. 7). We noticed that levels of sAPPα in the neocortex of DHT-lesioned mice decreased significantly compared with sham-operated APP/PS1 mice (*p* < 0.05) (Fig. 7a), whereas no changes were detected in the hippocampus (Fig. 7b). Levels of sAPPβ in the neocortex and hippocampus of sham-operated and DHT-lesioned APP/PS1 mice did not differ between groups (Fig. 7c, d).

## Discussion

Recently, it was reported that SSRI treatment attenuates plaque pathology in the APP/PS1 mouse model of familial AD [24, 32, 33]. To further test this hypothesis, we treated 3-month-old APP/PS1 mice with the more selective SSRI escitalopram [34] for a period of 6 months at a dosage of 5 mg/kg/day. This dosage was predicted to inhibit SERT in the therapeutic range, as confirmed by a SERT occupancy assay. We did not observe any changes in body weight, although this is a common side effect of SSRI treatment in both humans and rodents [35–37].

Levels of Aβ40 and Aβ42 were analysed in the CSF, neocortex and hippocampus. In the CSF of APP/PS1 mice, we observed no differences in the levels of Aβ40 and Aβ42 between escitalopram-treated and vehicle-treated APP/PS1 mice. Studies where human CSF has been analysed after orally administered SSRIs are ambiguous and show that although citalopram (2 × 30 mg) reduces the level of total Aβ in the CSF [32], escitalopram (2 × 20 mg) has no effect on levels of Aβ [38]. Analysis of the CSF of 3-month-old APP/PS1 mice that were treated for 4 months with orally administered citalopram (8 mg/kg/day) supports citalopram’s Aβ-reducing potential [24]. Results additionally suggested that citalopram’s Aβ-reducing effect was likely due to reduced production rather than increased clearance of Aβ [24, 32]. The reason for the absence of an SSRI effect in our study is unknown. It is possible that the effect on CSF levels of Aβ of this drug class depends on the type of SSRI used because escitalopram and citalopram, though very similar, show different effects in healthy human subjects. However, as a refinement of the study by Emilsson et al. [38], it was demonstrated that a single i.p. injection of 5 mg/kg escitalopram in 3- to 4-month-old APP/PS1 mice reduced interstitial levels of Aβ in the hippocampus [39]. Because the interstitial fluid under normal conditions is in equilibrium with the CSF, this observation at least shows that escitalopram can have effects, but it also suggests that some routes of administration may be more suitable than others.

In the neocortex of APP/PS1 mice treated with escitalopram, we observed a tendency toward an increase in the total content of insoluble Aβ compared with vehicle-treated mice, which is probably due to an increase in levels of Aβ40 and, to a lesser extent, Aβ42. We observed no changes in the levels of Aβ40 or Aβ42 in the hippocampus of escitalopram-treated compared with vehicle-treated mice. Our observations differ from recent clinical and in vivo studies in which researchers have reported Aβ-reducing effects of chronic and acute antidepressant treatment [24, 25, 32, 33, 40], although others have also been unable to detect such Aβ-reducing effects [38, 41]. Human subjects with a history of antidepressant drug treatment showed reduced cortical amyloid load compared with non-treated subjects, estimated by positron emission computed tomographic imaging using the amyloid-binding [^11^C]-Pittsburgh compound B (PIB) radioligand [24]. However, as the authors of that paper pointed out, a randomised clinical trial is needed to ascertain whether the observed reduction in PIB binding is indeed caused by SSRI treatment and not by past history of depression or anxiety. It has also been shown that 5 months of treatment with paroxetine i.p. (5 mg/kg/day) reduces Aβ40 levels in the hippocampus of 10-month-old 3 × Tg mice [25], as well as that 4 months of treatment with citalopram (8 mg/kg/day) per os reduces levels of Aβ and plaque load in the neocortex and hippocampus of 7-month-old APP/PS1 mice [24]. Treatment with citalopram (10 mg/kg/day) i.p. for 28 days has furthermore been shown to completely inhibit the growth of plaques without affecting their elimination [32]. Results at our own laboratory have suggested, however, that 9-month treatment with paroxetine (30 mg/kg/day being reduced to 10 mg/kg/day and ultimately 5 mg/kg/day) per os has no effect on plaque load in the neocortex of 18-month-old APP/PS1 mice [41], although a significant effect was observed in the hippocampus of the same mice [40]. Although these findings suggest that a high dosage of paroxetine may have an effect on plaque load at least in the hippocampus, they at the same time emphasise that paroxetine treatment by no means ameliorates plaque load when administered to aging mice in which the amyloid plaques are well-developed.

Escitalopram consists largely of an *S*-enantiomer, whereas citalopram used in the other studies is a racemic mixture of both *S*- and *R*-enantiomers in the ratio of 1:1. Several lines of evidence suggest that the *R*-enantiomer may counteract the effect of the *S*-enantiomer [42], leaving escitalopram more potent than citalopram. In our opinion, it is reasonable to assume that a dose of around 5 mg/kg/day of escitalopram would at least be equivalent to the effect of 8–10 mg/kg/day of citalopram, which is around the dose reported to effect amyloidosis in 7-month-old APP/PS1 mice [24, 32]. It should be noted, however, that Sheline et al. performed an experiment showing that a single i.p. injection of escitalopram (5 mg/kg/day) reduced the levels Aβ40 in the hippocampal interstitial fluid of APP/PS1 mice [39]. Therefore, it is not possible to completely rule out that escitalopram influences Aβ levels; however, its effect may depend on whether administration is acute or chronic, as well as on the route of administration. It is possible that the *R*-enantiomer present in citalopram may elicit Aβ-reducing effects independently of SERT [38], possibly through a direct influence on APP processing [43, 44]. The *R*-enantiomer has also been found to exert SERT-independent effects by acting on an orphan sigma-1 receptor (σ1, Oprs1), affecting axonal outgrowth and guidance in subpopulations of embryonic thalamocortical neurons [45, 46]. This being said, a single citalopram (10 mg/kg) i.p. injection in APP/PS1 mice was reported to reduce levels of Aβ40 in brain interstitial fluid, possibly through stimulation of α-secretase activity involved in the non-amyloidogenic pathway [24]. We therefore also investigated levels of sAPPα and sAPPβ, which are the first products of APP processing when cleaved by α- and β-secretase, respectively. Because we did not detect any differences in either sAPPα or sAPPβ protein levels, it is unlikely that the activities of these enzymes were affected by the escitalopram treatment in our study.

The APP/PS1 mice that were lesioned with the neurotoxin DHT were remarkably unaffected by the intervention and showed the same body weight gain as sham-operated mice, similar to what was reported in a previous study of shorter duration [27]. Plaques formed in the neocortex and hippocampus of APP/PS1 mice lesioned with the DHT and in sham-operated mice with similar propensity. Meso Scale Discovery multiplex analysis did not reveal any significant differences in soluble or insoluble fractions of Aβ40 or Aβ42 in the neocortex or hippocampus between DHT-lesioned and sham-operated mice. In a recent study where 7-month-old APP/PS1 mice were examined 2 weeks after DHT lesioning of the raphe, no changes in behaviour or Aβ pathology were detected either. Instead, the authors detected increased tau phosphorylation [27], which is not entirely unexpected, however, because tau phosphorylation increases during neuronal stress [47, 48]. We also looked into changes in the levels of sAPPα and sAPPβ to determine whether 5-HTergic deafferentation would impact the processing of APP. Interestingly, we found that levels of sAPPα were reduced in the neocortex of DHT-lesioned mice. We are not aware of any studies that have addressed whether α- or β-secretase is expressed predominantly in fibres or in somata, however, if α-secretase is mainly expressed in the 5-HTergic terminals, it would be logical to assume that deafferentation would reduce its presence. Reduction in the levels of the α-secretase cleavage product sAPPα were also independent of production of sAPPβ as well as production of Aβ. Overall, deafferentation of cortical 5-HT fibres did not affect levels of Aβ peptides, but it did, however, cause reduced levels of sAPPα, the biological significance of which is uncertain.

## Conclusions

Our observations do not support chronic escitalopram treatment as a strategy for anti-Aβ therapy, because we observed significant increases in the levels of insoluble Aβ in the neocortex of APP/PS1 mice. 5-HTergic deafferentation of the cortex did not affect the production of Aβ peptides. However, we did observe a reduction in sAPPα levels. Overall, our findings do not support modulation of the 5-HTergic system with the purpose of reducing Aβ levels in prodromal and early AD.

## Additional files

Additional file 1: Figure S1. Pilot study on the escitalopram treatment experiment. **a**, **b**, **c** In a preliminary escitalopram treatment experiment (**a**), concentration was closely monitored for a period of 4 weeks, and intake was calculated on the basis of body weight (**b**) and water intake (**c**). The average dosage of 3.83 ± 0.2 mg/kg/day is represented by the *dashed line*. (PNG 717 kb)

Additional file 2: Figure S2. Validation of the mesoscale analysis. Levels of total Aβ were measured in the (**a**) insoluble (guanidine) and (**b**) soluble (PBS) fractions in the neocortex of 6-month-old wild-type (*n* = 2), and 6- and 9-month-old APP/PS1 mice (*n* = 12–18). Data are presented as mean ± SEM and were analysed by unpaired, two-tailed Mann-Whitney *U* test. **p < 0.01, ***p < 0.001. (PNG 1888 kb)

## Abbreviations

Aβ: Amyloid-β
AD: Alzheimer’s disease
APP: Amyloid precursor protein
APP/PS1: APP_SWE_/PS1_ΔE9_
CSF: Cerebrospinal fluid
[^3^H]DASB: 3-Amino-4-(2-dimethylaminomethylphenylsulfanyl)-benzonitrile
DHT: 5,7-Dihydroxytryptamine
5-HIAA: 5-Hydroxyindoleacetic acid
HPLC: High-performance liquid chromatography
5-HT: Serotonin
5-HTergic: Serotonergic
O/N: Overnight
PFA: Paraformaldehyde
PIB: [^11^C]-Pittsburgh compound B
RT: Room temperature
sAPP: Soluble amyloid precursor protein
SB: Sørensen’s Buffer
SERT: Serotonin transporter
SSRI: Selective serotonin reuptake inhibitor
TBS: Tris-buffered saline
TBS-T: Tris-buffered saline with 1% Triton X-100
3 × Tg: Triple transgenic
