# Supplementary figures and images for: Serotonin augmentation therapy by escitalopram has minimal effects on amyloid-β levels in early-stage Alzheimer’s-like disease in mice
Source: Alzheimers Res Ther. 2017 Sep 12;9:74. doi: 10.1186/s13195-017-0298-y (PMC5596844; doi:10.1186/s13195-017-0298-y)

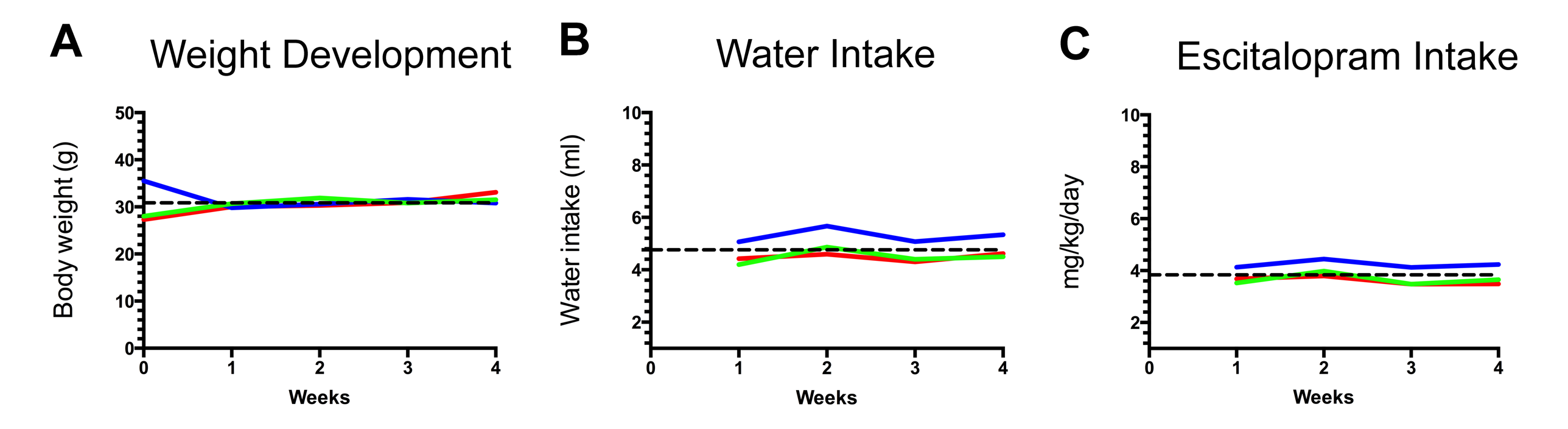

Supplement: Supplementary file 1 — Pilot study on the escitalopram treatment experiment. a, b, c In a preliminary escitalopram treatment experiment (a), concentration was closely monitored for a period of 4 weeks, and intake was calculated on the basis of body weight (b) and water intake (c). The average dosage of 3.83 ± 0.2 mg/kg/day is represented by the dashed line. (PNG 717 kb) [file 13195_2017_298_MOESM1_ESM.png]

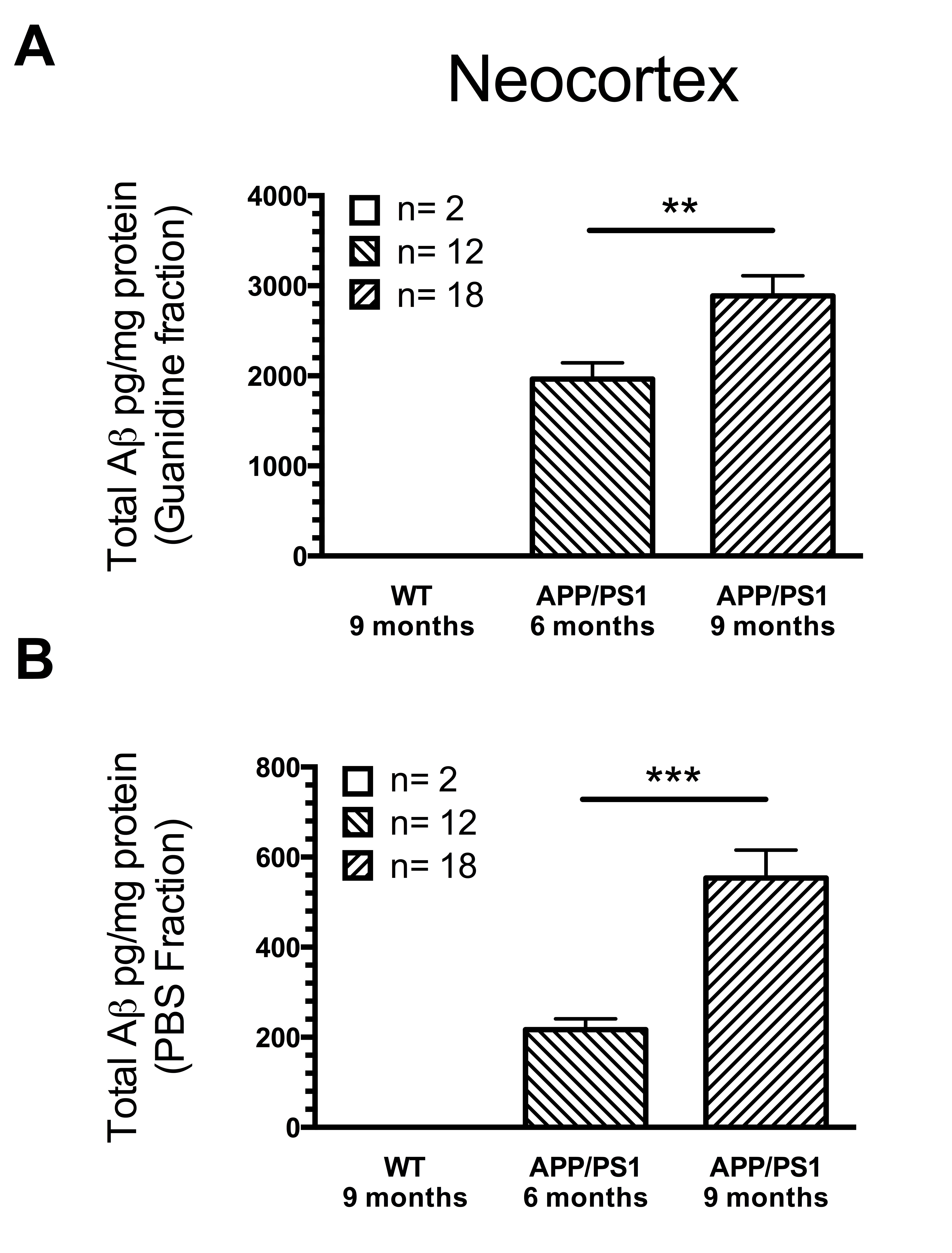

Supplement: Supplementary file 2 — Validation of the mesoscale analysis. Levels of total Aβ were measured in the (a) insoluble (guanidine) and (b) soluble (PBS) fractions in the neocortex of 6-month-old wild-type (n = 2), and 6- and 9-month-old APP/PS1 mice (n = 12–18). Data are presented as mean ± SEM and were analysed by unpaired, two-tailed Mann-Whitney U test. **p < 0.01, ***p < 0.001. (PNG 1888 kb) [file 13195_2017_298_MOESM2_ESM.png]
